# A study on comparison of overall survival and disease- free survival among gastric cancer patients treated with two adjuvant and neoadjuvant methods

**Published:** 2015

**Authors:** K Saadati, M Moghimi, S Baba Ali, R Eghdam Zamiri

**Affiliations:** * Department of Surgery, Mousavi Hospital, Zanjan, Iran; ** Department of Hematology Valiasr Hospital-Zanjan, Iran; *** University of Medical Science, Zanjan, Iran

**Keywords:** gastric cancer, adjuvant method, neoadjuvant method

## Abstract

The optimal management of locally gastric cancer persists a matter of intense discussion. Frequently cases with esophagogastric cancer are handled with preoperative chemotherapy [the more typical European method] or mixed chemoradiotherapy. The present research examines a comparison of overall retention and disease-free retention among gastric cancer cases managed via two Adjuvant and Neoadjuvant methods. We showed the features of quick gastric neoplasms operated by ESD. This research showed that ESD for quick gastric neoplasms is a typical approach since the en bloc and curative resection percentages are very high, and residual infection or recurrence is limited. Nevertheless, we further demonstrated that the obstacles connected to this method are the long method time and comparatively high rates of procedure-related developments. We should explore methods to reduce the method time and reduce these difficulties.

## Introduction

Gastric cancer being the second principal cancer reason in the societies [**14**], gastric cancer is the most malignancy specified in South America, Japan and Eastern Europe [**1**,**4**]. The overall prevalence of gastric cancer seems to decrease in the world. However, the new cases are still increasing year by year which may be due to the rise in the average lifetime of the generations. The prevalence of gastric cancer differs in different geographical areas. So we could divide the world into three subdivisions: high risk areas (Age-Adjusted Rate, ASR > 20) including China, Russia, Japan, Chile, Costa-Rica, Korea and Ecuador; regions with intermediate risk (ASR > 10) including Eastern Europe, Mexico, Brazil and India and the areas with low risk (ASR < 10) including the United State America, Western Europe, Australia, Canada and Indonesia [**2**]. In Iran, there is a lot of difference in gastric cancer incidence between distinct zones [**5**].

Most north and north areas of Iran are at a large risk of gastric cancer [**3**]. The Northern regions especially the Azeri provinces are in a high-risk group, the western and central parts are in the intermediate risk group, and the south areas are among low-risk areas. There are two types of classifications for gastric cancer, anatomical and histological. Unlike the European Western countries, Northern America and Japan, the prevalence of gastric cancer had an increasing trend in Iran in the past 30 years, and it has reached the peak in Ardebil, Azerbaijan [**2**]. Example major question to be asked is that the greater prevalence ratio of gastric cancer in Ardabil is since of the greater ratio of gastric cardia preferably than non-cardia cancer, remaining 26 and 9 in males and females. Each which is opposed to another great - danger zone i.e. Japan, where non-cardia cancer persists as an important role in gastric cancer [**3**].

The right primary treatment for gastric cancer is surgery at resectable stages while chemotherapy could modify the prognosis of cases, however, via complete resection and without distal metastasis, it still is an aggressive disease with poor prognosis. Some patients being in levels II (excluding T1 problems) and III (mean advanced) have shown recurrence after curative resection [**6**,**11**,**13**,**15**].

According to Xin-Zu et al. 2011, neoadjuvant chemotherapy is a proper treatment to advance the R0 resection ratio for local adenocarcinoma gastric cancer. However, mixed neoadjuvant and adjuvant would modify the overall retention of the cases [**6**-**12**,**16**]. These findings were parallel via the meta-investigation conducted by Li [**17**]. 

According to the personal information of 3838 cases of seventeen 17 various trials via a medium follow-up larger than seven years, the largest case-stage meta-investigation conducted so far, we noted a moderate however statistically important profits linked with adjuvant chemotherapy later gastric cancers curative resection. 

According to a randomized trial conducted in 2001, an obvious advance was seen in medium retention of nine months in patients who had chemoradiotherapy after surgery. On the other hand, we have the neo-adjuvant trials, in which a benefit of starting chemotherapy as soon as possible reached. Since more patients can take neo-adjuvant chemotherapy, it obtained acceptance over the western areas [**18**].

Finally, according to the ambiguous results from numerous phase3 researches containing a surgery-only team, the effectiveness of adjuvant chemotherapy is problem. Hence, nominal administration following curative operation is heterogeneous though the various areas.

## Materials and methods

A whole amount of 79 cancer cases from Zanjan involved in current research. These cases mostly associated with the Vali-e-Asr Hospital since 2007 and were followed until 2014. Our aim was to perform end so no on patients, however, since the facilities were not possible we used only CT scan and endoscopy for staging.

All the 79 patients referred to the Vali-e-Asr Hospital were suspected of having gastric cancers. The patients with metastasis and ascites excluded from this study. In a Neoadjuvant group, laparoscopy before surgery has been considered for the patients to reject involvement with peritoneum.

The patients with gastric cancer classified into gastric cardia adenocarcinoma (GCA) and gastric non-cardia adenocarcinoma (GNCA). Adenocarcinoma of the stomach categorized as an intestinal or diffuse kind application Lauren’s categorizing standard (Lauren, 1965). Patients in the adjuvant group treated with chemoradiation protocol (INT-0116), and one Uncovered SF course and the Neoadjuvant group received one course ECF diet, surgery and then another course of ECF menu. In chemoradiation group, radiotherapy 45 Gy and chemotherapy (Xeloda) with a dose of 62 mg per square meter has been used. 

## Result

In the present research, most of the participants consist of males (78%). The patients under adjuvant diet therapy consist of 72% men and the patients under Neoadjuvant diet therapy consist of 72.5% males. In this case, two groups are similar to each other. 

In general, among all the patients included in the present research, the majority of patients (67%) had T staging III disease and patients with me -T stage has mentioned as the least number of clients (2% & 3%). Among the patients under treatment with adjuvant diet, 73% and 3% had III -T stage and I & IV -T stage, respectively. This rate has been reported about 54% among the patients who received Neoadjuvant diet and 3% among the patients in the groups with I & IV -T stage, found without a significant difference. Further, a majority of the patients (70%) who had the conditions for inclusion in this study had the tumors with grade II. The least patients (9%) with the conditions for inclusion in this study had the tumors with grade I. Similarly, the patients under adjuvant (85%) and Neoadjuvant (53%) diets were the patients with grade II.

The average age has been 63 years old, and average DFS (Disease Free Survival) has been 12.3 months. The rate of DFS in patients in the groups under study in both Adjuvant and Neoadjuvant groups has been 11.36 and 13.28 months, indicating the higher rate of this index in a Neoadjuvant group than the adjuvant group. The average age in Adjuvant and Neoadjuvant groups has been 61 and 65 years old, respectively. Concerning the obtained results, the median age has been greater in the Neoadjuvant group than an adjuvant group, found without a significant difference. The results from this study indicated that rate of overall survival in an adjuvant group has been about 18.5 months in a seven years follow-up, which this rate has been about 17.6 months in the Neoadjuvant group, found without a significant difference. In this study, 22.2% of the patients have been under R1 surgery with the positive margin, yet 77.8% of the patients have been under R0 surgery, mentioned that ratio of R0 reporting has been larger in the Neoadjuvant team. Due to lack of suitable lymph node dissection, the cases classified into 2 teams LN+(71.4%) and LN-(28.6).

## Discussion

In the present research, 79 patients were examined in two separate groups; a group consists of the patients (39) who received adjuvant diet therapy, and another group consists of the patients (40) who received Neoadjuvant diet therapy. Most of the cases involved in the study consist of the males, and the average age of the clients have been 63 years old, considered in two adjuvant (61 years old) and Neoadjuvant (65 years old) groups, indicating greater average age in the Neoadjuvant group. There has been the same status of grade and T staging among the patients under study in both Adjuvant and Neoadjuvant groups so that majority of patients had III T stage and II class. According to a study by Christoph Schumacher et al. (2009), they failed to find an advantage for chemotherapy via Neoadjuvant method compared to adjuvant treatment, which this can confirm the findings from the present research. In a meta-analysis by Paoletti X et al. (2010), it has been indicated that chemotherapy via adjuvant method is useful in treating stomach cancer, which these results do not confirm the findings from the present research. Concerning the most extensive study which has conducted to date by GASRRIC GROUP (2010) in which the patients have been followed up for seven years, chemotherapy via adjuvant method outperforms surgical resection, found with numerous benefits for the patients. Since receiving adjuvant method among the patients with stomach cancer is more common in western countries, Neoadjuvant treatment was considered superior to adjuvant treatment for the patients. In another study by Mitsuru Sasako et al. (2011) to examine the usefulness of adjuvant chemotherapy to stomach resection, satisfactory results about the effectiveness of receiving adjuvant treatment have not found. The studies on various meta-analyses indicated that receiving adjuvant diet therapy has been more useful than stomach resection for the patients, which these findings confirmed in the present research. Concerning a review on stomach resection surgery in after-surgery survival and receiving adjuvant diet therapy in western countries in 2010, GASTRIC group indicated that R0 resection surgery with adjuvant diet therapy was superior to surgery, such that survival of patients who have undergone surgery reaches to 16.4 months. In another study by Xin ZU et al. (2011), treatment with chemotherapy via Neoadjuvant treatment has increased among the patients who have undergone R0 resection surgery. Combined Adjuvant and Neoadjuvant treatment have been followed by an increase in survival among the patients, which these results are consistent with the results of research by Li et al. (2010). What seen in this study lies in increasing rate of Lymph nodes among the patients treated with Neoadjuvant treatment, resulted in worsening the prognosis of patients; further, most of the patients with negative Lymph nodes have been in the adjuvant group. Therefore, concerning no difference on DFS among the patients treated with adjuvant and neoadjuvant patients, this might be due to lack of uniform division of patients with Lymph node involvement. This study shows that there is no significant difference on DFS between Adjuvant and Neoadjuvant groups, which it must repeat with the greater number of patients. 
